# Oxidized low-density lipoprotein (oxLDL) affects load-free cell shortening of cardiomyocytes in a proprotein convertase subtilisin/kexin 9 (PCSK9)-dependent way

**DOI:** 10.1007/s00395-017-0650-1

**Published:** 2017-09-14

**Authors:** Klaus-Dieter Schlüter, Annemarie Wolf, Martin Weber, Rolf Schreckenberg, Rainer Schulz

**Affiliations:** Institute of Physiology, Justus-Liebig-University Giessen, Aulweg 129, 35392 Giessen, Germany

**Keywords:** Hypercholesterolemia, Heart failure, Oxidative stress

## Abstract

Recent studies have documented that oxidized low-density lipoprotein cholesterol (oxLDL) levels directly impact myocardial structure and function. However, the molecular mechanisms by which oxLDL affects cardiac myocytes are not well established. We addressed the question whether oxLDL modifies load-free cell shortening, a standardized readout of cardiac cellular function, and investigated whether proprotein convertase subtilisin/kexin-9 (PCSK9) is involved on oxLDL-dependent processes. Adult rat ventricular cardiomyocytes were isolated and incubated for 24 h with oxLDL. PCSK9 was silenced by administration of siRNA. Load-free cell shortening was analyzed via a line camera at a beating frequency of 2 Hz. RT-PCR and immunoblots were used to identify molecular pathways. We observed a concentration-dependent reduction of load-free cell shortening that was independent of cell damage (apoptosis, necrosis). The effect of oxLDL was attenuated by silencing of oxLDL receptors (LOX-1), blockade of p38 MAP kinase activation, and silencing of PCSK9. oxLDL increased the expression of PCSK9 and caused oxidative modification of tropomyosin. In conclusion, we found that oxLDL significantly impaired contractile function via induction of PCSK9. This is the first report about the expression of PCSK9 in adult terminal differentiated ventricular cardiomyocytes. The data are important in the light of recent development of PCSK9 inhibitory strategies.

**Electronic supplementary material:**

The online version of this article (doi:10.1007/s00395-017-0650-1) contains supplementary material, which is available to authorized users.

## Introduction

Oxidative stress defines a condition in which the formation of reactive oxygen species (ROS) exceeds the oxidant defense capacity of cells. Low-density lipoprotein (LDL) particles are susceptible to oxidation and hence the plasma levels of oxidized LDL (oxLDL) particles increase under conditions of oxidative stress. Oxidative stress is a hall mark of several diseases including cardiovascular disease. oxLDL participates in the progression of disease under condition of oxidative stress. In line with these suggestions, plasma oxLDL levels seem to be a predictor for mortality in patients with chronic heart failure suggesting that oxidative stress is causally involved [31]. However, there are only very limited data analyzing the effect of oxLDL on cardiomyocytes.

Of note, plasma levels of oxLDL have been associated with a decrease in cardiac function independent of vascular alterations [21]. Furthermore, oxLDL induced apoptosis in neonatal rat cardiomyocytes [8], it prolonged action potential and it depolarized resting potential in rabbit cardiomyocytes [36]. OxLDL induced the expression of biomarkers associated with heart failure in HL-1 cells, an immortalized cardiac-like cell type [2]. On the other hand, oxLDL increased calcium transients in rabbit cardiomyocytes [15] and it improved L-type currents in rat ventricular cardiomyocytes [5]. In most cases, the effects of oxLDL could not be mimicked by LDL [2, 5, 36]. This suggests that oxidative stress first modifies LDL particles that then secondarily affect cardiomyocytes.

Nevertheless, there are also reports that suggest that LDL can affect cardiac function independent of oxidative modification [1, 9, 14]. Furthermore, activation of the classical oxLDL receptor, lectin-like oxidized low-density lipoprotein receptor 1 (LOX-1), has been linked to cross-activation of angiotensin II receptor pathways [10, 11, 16, 29, 33]. Finally, cardiomyocytes represent a specific regulation of cholesterol homeostasis independent of LDL-receptor traffic, suggesting that the different receptors play mainly signaling roles rather that they represent transport pathways [20]. These examples clarify that the role of LDL, oxLDL, and LOX-1 activation for cardiac function remains unclear.

The question whether oxLDL affects ventricular cardiomyocytes is not fully understood and complicated by rather controversial findings as explained above. Here, we used a well-established model of cultured adult rat ventricular cardiomyocytes that can easily be analyzed within 24 h under serum-free conditions and therefore under fully controlled conditions. We used this setup to study the effect of oxLDL on ventricular cardiomyocytes and specifically to address the question whether proprotein convertase subtilisin/kexin-9 (PCSK9) is involved in this reaction. PCSK9 is a recently identified modulator of LDL and oxLDL receptor turnover [25]. Moreover, oxLDL induces the expression of PCSK9 in non-hepatic cells and PCSK9 is required to trigger oxLDL-dependent activation of NFκB-pathways [7, 27]. However, it remains elusive whether it is expressed in cardiomyocytes and whether it plays a role in oxLDL-dependent effects on this cell type. PCSK9 inhibition is an upcoming therapeutic target for patients with hypercholesterolemia and due to the high coincidence of heart failure in this population, it is important to understand the role of oxLDL, LOX-1, and PCSK9 in cardiomyocytes.

## Materials and methods

### Materials

oxLDL (human high oxidized low-density lipoprotein) was produced by KB Kalen Biomedical, Montgomery Village, USA and purchased from Biotrend Chemikalien, Cologne, Germany. oxLDL stock solution was stored at 4 °C and used within 4 weeks. Actinomycin D, cycloheximide, PD98059, SB202190, and SP600125 were purchased from Merck KGaA (Darmstadt, Germany). Secondary antibodies directed against rabbit IgG and mouse IgG were purchased from Sigma-Aldrich (Taufkirchen, Germany).

### Isolation and cultivation of cardiomyocytes

Four-month-old male Wistar rats were used. Rats were housed according to the Guide for the Care and Use of Laboratory Animals (NIH Publication No. 85-23, revised 1996). All protocols were approved by the Justus-Liebig-University Giessen (permission number: 559_M). Ventricular heart muscle cells were isolated from rats as described previously and used routinely in the group [22]. Briefly, hearts were excised under deep anesthesia, transferred rapidly to ice-cold saline, and mounted on the cannula of a Langendorff perfusion system. Hearts were perfused in a non-circulating mode with a calcium-free perfusion buffer and then in recirculating mode adding collagenase (type 2 CLS 2 270 U/mg, Worthington) with CaCl_2_ (25 µM) for 25 min. Thereafter, ventricular tissue was minced and incubated for another 5 min in recirculating buffer. The remaining cell solution was filtered through a 200-µm nylon mesh. The filtered material was resuspended in buffer with a stepwise increase in calcium and finally transferred to culture medium (Medium 199, supplemented with creatine, carnitine, taurine, and 2% penicillin–streptomycin, Biochrom). Cells were plated to Petri dishes which were precoated with 4% (v/v) FCS (PAA, BioPharm) and penicillin–streptomycin (Gibco, Thermo Fisher Scientific) in culture medium for 1 h. Thereafter, cell culture medium was refreshed and cells were used for subsequent analysis or further incubated for 24 h at 37 °C.

### Cell culture regimes

Cells were cultured for 24 h in the above mentioned medium under serum-free conditions. Where indicated, small inhibitory RNA (siRNA) directed against LOX-1, PCSK9, or scrambleRNA (scrRNA were supplied 6 h before administration of oxLDL (final concentration 0.05 µM). siRNA was purchased from Qiagen, the Netherlands. Inhibitors of MAPK pathways (10 µM) were used as previously shown to act in the same experimental system [35]. Actinomycin D (5 µM) and cycloheximide (35 µM) were used as previously shown [23].

### Cell shortening

Cells were stimulated via two AgCl electrodes with biphasic electrical stimuli composed of two equal but opposite rectangular 50-V stimuli of 5-ms duration as described before [12]. Cells were stimulated with 2 Hz frequency. Four signals were registered from each cell. The mean of these four measurements was used to define the contractile responsiveness of a given cell. Cell lengths were measured at a rate of 500 Hz via a line camera. Cells were used in M199 with an extracellular calcium concentration of 1.25 mM. Data are expressed as Δ*L*/*L* (%) in which the shortening amplitude (Δ*L*) is expressed as percent of the diastolic cell length (*L*). Furthermore, maximal contraction, relaxation velocity, time to peak 50% of contraction, time to peak of contraction, and time to reach 50% of relaxation were analyzed.

### Apoptosis and necrosis

Quantification of the number of cells that lost cell viability by necrosis was quantified as previously shown [28]. Cells were stained with propidium iodide (1 µg/ml). Viable cells are able to extrude the dye. Dead cells are indicated by red fluorescence when excited at 510–550 nm. Apoptotic cells were identified by supplementation of HOE33258 (5 µg/ml) to the cells. Apoptotic cells were identified by clear nuclear chromatin condensation.

### Western blots

Isolated cardiomyocytes were incubated with lysis buffer as described before [24]. Samples (≈100 μg protein dissolved with 10 µl of bromphenol blue) were loaded on a 10% SDS-PAGE and blotted onto membranes. Anti-PCSK9 (rabbit polyclonal, ab31762, Abcam, Cambridge, UK), anti p38 MAPK (rabbit polyclonal, #506123, Merck KGaA Darmstadt, Germany), and anti phosphorylated p38 MAPK (rabbit polyclonal, #M0800, Sigma, St. Louis, USA) antibodies were used to detect the expression of PCSK9 protein and phosphorylation of p38 MAPK. Anti-LOX-1 (rabbit polyclonal, #GTX59636, GeneTex, USA) antibody was used to analyze the expression of LOX-1. Glyceraldehyde 3-phosphate dehydrogenase (GAPDH) was used as loading control. Detection of GAPDH was performed using anti-GAPDH monoclonal antibody produced in mouse (CB1001, Merck, Germany).

Oxidative modification of tropomyosin was performed as shown before [24]. Briefly, protein from cardiomyocytes was extracted using cell lysis buffer (50 mM Tris, pH 6.7, 2% v/v SDS, protease and phosphatase inhibitors; purchased from Cell Signaling). 100 µl of these samples was diluted with 40 µl of non-reducing Laemmli buffer (Boston Bio Products, BP-MONR) and samples were loaded on a gel under non-reducing conditions. After transfer on nitrocellulose membranes, tropomyosin was identified with anti-Tm (Sigma-Aldrich, T9283) as primary antibody and horseradish peroxidase-coupled secondary antibody. Controls were performed under reducing conditions using the same lysis buffer but with 50 mM DTT and samples were diluted with Laemmli buffer for reducing probes (Sigma-Aldrich, S3401).

### ELISA

Release of PCSK9 from cardiomyocytes was quantified by the use of a commercial available kit purchased by Cusabio Biotech Co., China (Rat PCSK9 ELISA kit) and used following the instruction.

### qRT-PCR

Isolated cells were collected for PCR analysis using PBS cold solution. Total RNA from cardiomyocytes was extracted with Trizol (Invitrogen) as described by the manufacturer. After conversion of RNA into complementary DNA (cDNA) with reverse transcriptase, PCR was performed. Primers for PCSK9, bcl-2, bax, LOX-1, LDL-R, LRP-1, B2 M or GAPDH were used (Table 1). For control experiments, liver tissue was removed from the rats used for the isolation of cardiomyocytes. Tissues were quickly frozen into fluid nitrogen and RNA was extracted using peqGold TriFast (peqlab, Biotechnology GmbH, Erlangen, Germany) according to the manufacturer’s protocol. To remove genomic DNA contamination, isolated RNA samples were treated with 1 U DNase per mg RNA (Invitrogen, Karlsruhe, Germany) for 15 min at 37 °C. Real-time PCR was performed using iCycler iQ detection system (Bio-Rad, Munich, Germany) in combination with IQ SYBR green real-time supermix. Primer efficiency data were acquired by analysis of amplification curve using quantitative real-time PCR (iQ5, Biorad, USA) as described previously [32].

**Table 1:** List of primers used in this study

Gene	Forward	Reverse
B2M	GCCGTCGTGCTTGCCATTC	CTGAGGTGGGTGGAACTGAGAC
HPRT	CCAGCGTCGTGATTAGTGAT	CAAGTCTTTCAGTCCTGTCC
GAPDH	CTT CTC TTG TGA CAA AGT GGA CA	CTC GCT CCT GGA AGA TGG TG
Bcl-2	ATC TTC TCC TTC CAG CCT GA	TCA GTC ATC CAC AGA GCG AT
Bax	ACT AAA GTG CCC GAG CTG ATC	CAC TGT CTG CCA TGT GGG G
PCSK9	TTG AAC AAA CTG CCC ATC GC	CCC AAC AGG TCA CTG CTC AT
LOX-1	GGCCATCCTTTGCCTAGTGT	ACATCTGCCCCTCCAGGATA
LDL-R	CTGGCGGCTGAGGAACATTA	ATCCTCCAGGCTGACCATCT
LRP-1	GCGGTGTGACAACGACAA	GTCTTGTGGCCTGGTTGGTA

### Statistics

Data are expressed as raw data points or means ± SD as indicated in the legend to the figures. ANOVA and the Student–Newman–Keuls test for post hoc analysis were used to analyze experiments in which more than one group was compared. Normal variation of samples was verified prior to testing (Levene’s test). *p* levels are indicated as expressed in the legend to the figures or as an asterisk if *p* < 0.05. Comparison of two groups was performed by two-side *t* test or Mann–Whitney *U* test if appropriable, depending on the distribution of the samples.

## Results

### Effect of oxLDL on load-free cell shortening of cardiomyocytes

**Fig. 1:**
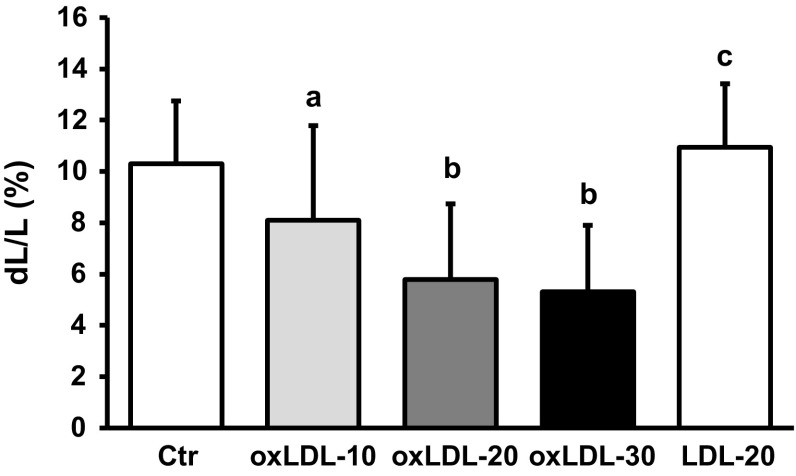
Effect of oxLDL on load-free cell shortening. Serum-free cultured adult rat ventricular cardiomyocytes were exposed to oxLDL with 10, 20 and 30 µg/ml oxLDL, respectively (oxLDL-10; oxLDL-20; oxLDL-30), for 24 h. Another group of cells was exposed to LDL (20 µg/ml). Load-free cell shortening is expressed as d*L*/*L* (%) of 117 (oxLDL-10), 162 (oxLDL-20), 36 (oxLDL-30), 135 (LDL-20), and 378 (control) cells from 13, 18, 15, 5, and 42 independent experiments, respectively. Cells were paced at 2 Hz. Data are mean ± SD. *a* *p* < 0.05 vs. control, oxLDL-20, and LDL-20. *b* *p* < 0.05 vs. control, oxLDL-10, LDL-20. *c* *p* < 0.05 vs. control, oxLDL-10, oxLDL-20, and oxLDL-30

The main important function of cardiomyocytes is to generate force by contraction. We therefore studied the effect of oxLDL on load-free cell shortening as readout of basal cardiac function. Serum-free cultured adult rat ventricular cardiomyocytes were incubated with oxLDL for 24 h. Thereafter, cells were paced at 2 Hz and load-free cell shortening was quantified as percent shortening amplitude normalized to the diastolic cell length of individual cells. oxLDL caused a concentration-dependent decrease of cell shortening that reached a maximum at 20 µg/ml (Fig. 1). Of note, this effect of oxLDL could not be mimicked by non-oxidized LDL (Fig. 1). Diastolic cell lengths were not affected by oxLDL (Table 2). Similar, time to reach 50% of peak shortening (TTP50) was not affected indicating no alterations in the initiation of electromechanical coupling (Table 2). However, time to peak (TTP) was shortened. This parameter depends on either maximal contraction velocity or the absolute shortening amplitude. In case of oxLDL, shortening of TTP was associated with reduced maximal contraction velocity and prolonged TTP when normalized to shortening amplitudes indicating reduced contraction dynamics. Similarly, time to reach 50% of relaxation (R50) was shortened and this was accompanied by reduced maximal relaxation velocity and prolonged R50 normalized shortening amplitudes (Table 2).

**Table 2:** Effect of oxLDL (10 µg/ml) on load-free cell shortening

	Control	oxLDL	*p* value
*n*	126	117	n.d.
L Diast (µm)	99.89 ± 16.56	99.38 ± 16.64	0.81
Amplitude (µm)	10.85 ± 2.86	8.14 ± 4.05	≤0.00
d*L*/*L* (%)	10.89 ± 2.33	8.10 ± 3.69	≤0.00
TTP50 (ms)	64 ± 10	63 ± 10	0.25
TTP (ms)	162 ± 27	151 ± 30	≤0.00
TTP/amplitude (ms/µm)	16.19 ± 6.52	25.71 ± 20.31	≤0.00
Contraction velocity (µm/s)	196 ± 59	155 ± 79	0.00
R50 (ms)	244 ± 36	233 ± 41	0.03
R50/amplitude (ms/µm)	24.48 ± 9.96	40.32 ± 33.14	≤0.00
Relaxation velocity (µm/s)	184 ± 68	152 ± 90	≤0.00

Data are mean ± SD; *n* = number of cells from three different rats (14 culture dishes)

*L Diast* diastolic cell length, *dL/L* shortening amplitude normalized to diastolic cell length (amplitude × 100/L Diast), *TTP* time to peak, *TTP50* time to reach 50% of peak shortening, *R50* time to reach 50% of relaxation

**Fig. 2:**
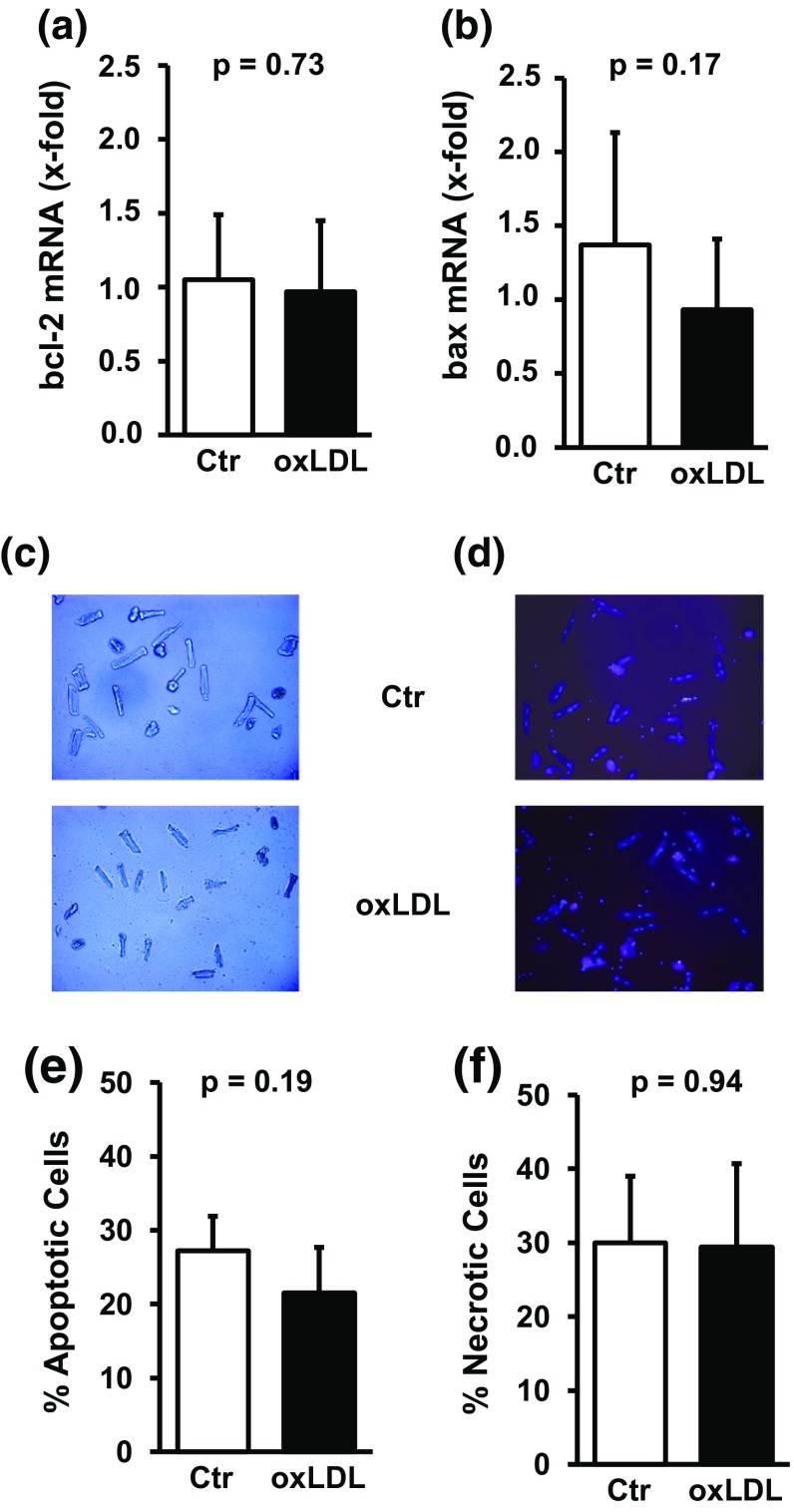
Effect of oxLDL on cell viability. Serum-free cultured adult rat ventricular cardiomyocytes were exposed to oxLDL (20 µg/ml) for 24 h. mRNA expression of bcl-2 (**a**) and bax (**b**). Data are mean ± SD from *n* = 9–10 cultures dishes (8 independent experiments). Light microscopy (**c**) and fluorescence overlay (**d**) from representative culture dishes. Percent of apoptotic (**e**) and necrotic cells (**f**) cells for controls and oxLDL-treated cultures (130 and 102 cells, respectively, from five independent experiments). Data are mean ± SD. Exact *p* values are given

The impairment of load-free cell shortening was not associated with general toxic effects of oxLDL on cardiomyocytes. oxLDL did not reduce the mRNA expression of the anti-apoptotic gene bcl-2 and oxLDL did not induce the expression of the pro-apoptotic gene bax (Fig. 2). Furthermore, no obvious morphological differences appeared including a similar number of rod-shaped cardiomyocytes (Fig. 2). The number of cells undergoing apoptosis or necrosis under cultured conditions did not differ between control cultures and those treated with oxLDL (Fig. 2).

### Effects of oxLDL depend on LOX-1 activation

**Fig. 3:**
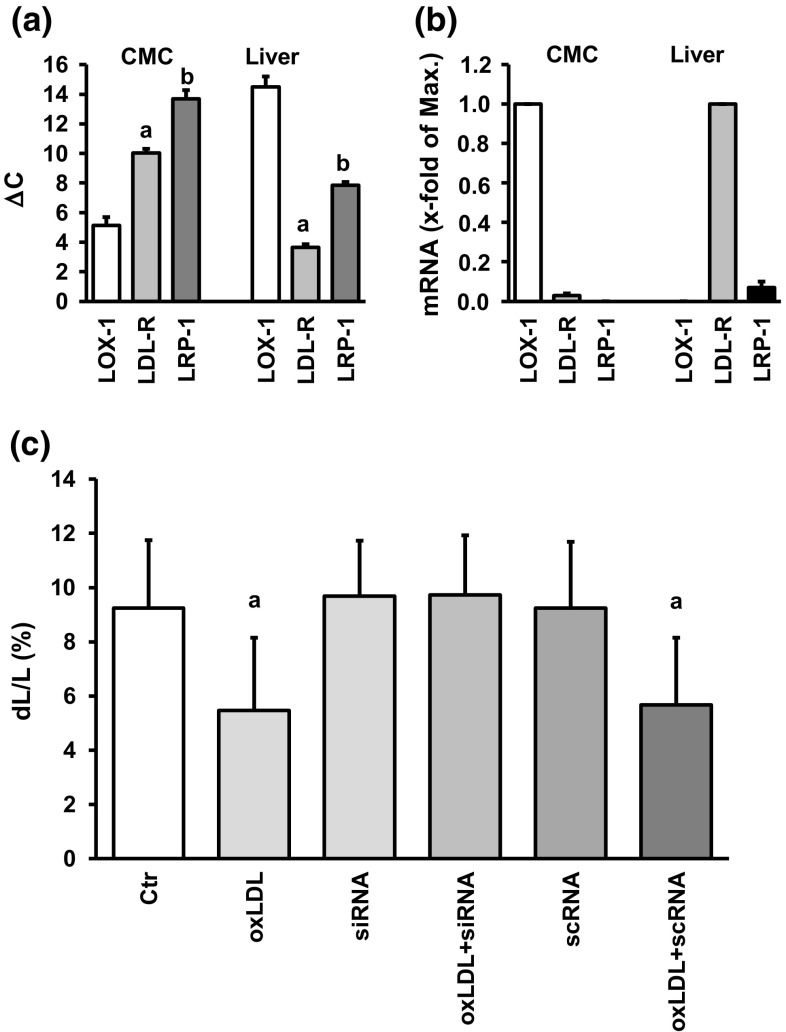
Expression of LDL receptors in cardiomyocytes (CMC) or liver. **a** Cycle number for the threshold of LDL receptor amplifications of samples from cardiomyocytes cultured for 24 h under control conditions or liver samples (*n* = 10, 8 independent experiments). Data are normalized to the mean expression of three house-keeping genes (HPRT, GAPDH, B2M). **b** Relative expression normalized to the expression of the strongest expressed receptor in each tissue. All data are normalized to the three house-keeping genes as an internal standard. **c** Effect of silencing of LOX-1 by supplying siRNA direct against LOX-1. oxLDL concentration was 20 µg/ml. ScrambleRNA represents a randomized sequence used as a control. Data are mean ± SD from 72 cells (nine independent experiments). a: *p* < 0.05 vs. control, siRNA, siRNA + oxLDL, and scrambleRNA

Cardiomyocytes poorly internalize exogenously supplied native and modified LDL [20]. Furthermore, they respond to oxLDL rather to LDL (Fig. 1). Therefore, we addressed the hypothesis that the above mentioned effects of oxLDL are triggered by an activation of LOX-1 [30]. First, we analyzed the expression of LDL receptor subtypes in adult ventricular cardiomyocytes. Primer efficiency was comparable between LOX-1, LDL-R, and LRP-1 (81.1, 82.1, and 81.8%, respectively). As indicated in Fig. 3, cardiomyocytes mainly express LOX-1. When analyzed by RT-PCR the amplification product was identified 5.2 ± 0.6 cycles later than house-keeping genes (mean threshold of HPRT, GAPDH and B2 M). The classical LDL receptor appeared 10.0 ± 0.3 cycles later (Fig. 3). LRP-1 was below the detection levels in most preparations and appeared rather late in the remaining samples (13.7 ± 0.6 cycles later). When normalized to the expression of LOX-1, LDL receptor expression was 3% of that of LOX-1 (Fig. 3). In comparison, LDL-R was by far the most dominant receptor subtype in the liver, with LOX-1 expression below the detection level in most liver preparations and very low expression in the remaining tissues (Fig. 3). As LOX-1 is, therefore, the most likely candidate that triggers the effects of oxLDL, a participation of LOX-1 was proven more directly by silencing LOX-1. siRNA directed against LOX-1 was supplied 6 h prior to stimulation with oxLDL. Whereas neither siRNA directed against LOX-1 alone nor scrambleRNA altered load-free cell shortening, the effect of oxLDL was significantly attenuated by silencing of LOX-1 with siRNA but not with scrambleRNA (Fig. 3). Compared to the total protein of the whole culture, i.e., including proteins of necrotic and apoptotic cells, mean expression of pro-LOX-1 in the presence of siRNA directed against LOX-1 was approximately 50% of that of cultures treated with scramble RNA (Supplement Fig. 1).

**Fig. 4:**
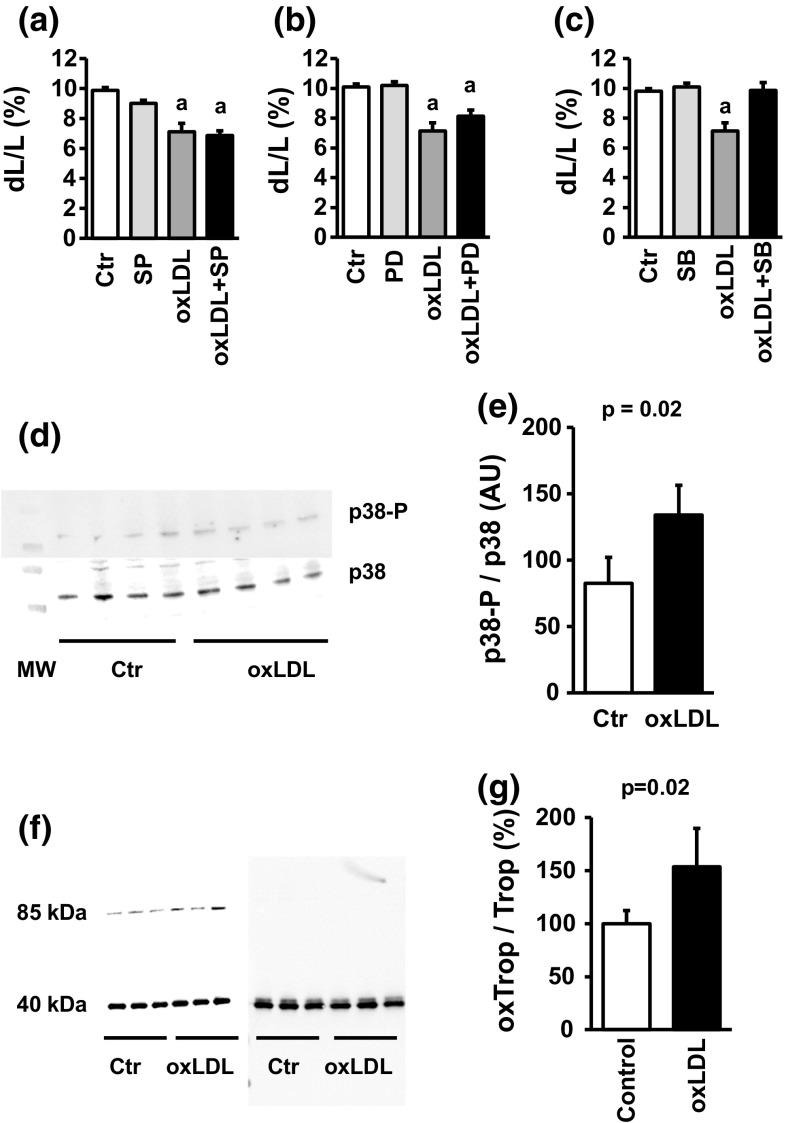
Participation of MAPK pathways in oxLDL-dependent effects: **a** cell shortening of cardiomyocytes exposed to oxLDL (20 µg/ml) and SP600125 (10 µM). **b** Cell shortening of cardiomyocytes exposed to oxLDL (20 µg/ml) and PD98059 (10 µM). **c** Cell shortening of cardiomyocytes exposed to oxLDL (20 µg/ml) and SB202190 (10 µM). In **a**–**c** a represents *p* < 0.095 vs. control. **d** Representative immunoblot of samples from cardiomyocytes exposed to oxLDL (20 µg/ml) and quantified for p38 MAPK expression and phosphorylation of p38 MAPK. **e** Quantification of the blot shown in **d**. **f** Original western blot showing the oxidative modification of tropomyosin by oxLDL (20 mg/ml) in the left and the control blot under reducing conditions (right). **g** Quantification of oxidative modification of tropomyosin by oxLDL (*n* = 3). Exact *p* values are given

Stimulation of LOX-1 has been linked to the activation of mitogen-activated protein kinase (MAPK) pathways in several experimental systems. The most consistent activation found in cells that respond to oxLDL is p38 MAPK, but there are also reports that oxLDL can activate p42/p44 MAPK and c-jun-kinase pathways [11, 26]. We first analyzed whether oxLDL-dependent effects are modified by inhibition of one of the MAPK pathways. The oxLDL-dependent effect on cell shortening was attenuated by SB202190, a p38 MAPK inhibitor, but nor by SP600125 or PD98059 that inhibit the two other MAPK pathways (Fig. 4a–c). Furthermore, oxLDL significantly increased the phosphorylation of p38 MAPK (Fig. 4d, e; Supplement Fig. 2). p38 MAPK is a stress-activated type of MAPK that is activated by oxidative stress [29]. Oxidative stress can also directly affect cell shortening by oxidative modification of tropomyosin [20]. Indeed, oxLDL increased the level of oxidative modified tropomyosin as indicated by a shift in the apparent molecular weight from 40 to 85 kDa (Fig. 4f, g; Supplement Fig. 3).

### Effects of oxLDL on PCSK9

**Fig. 5:**
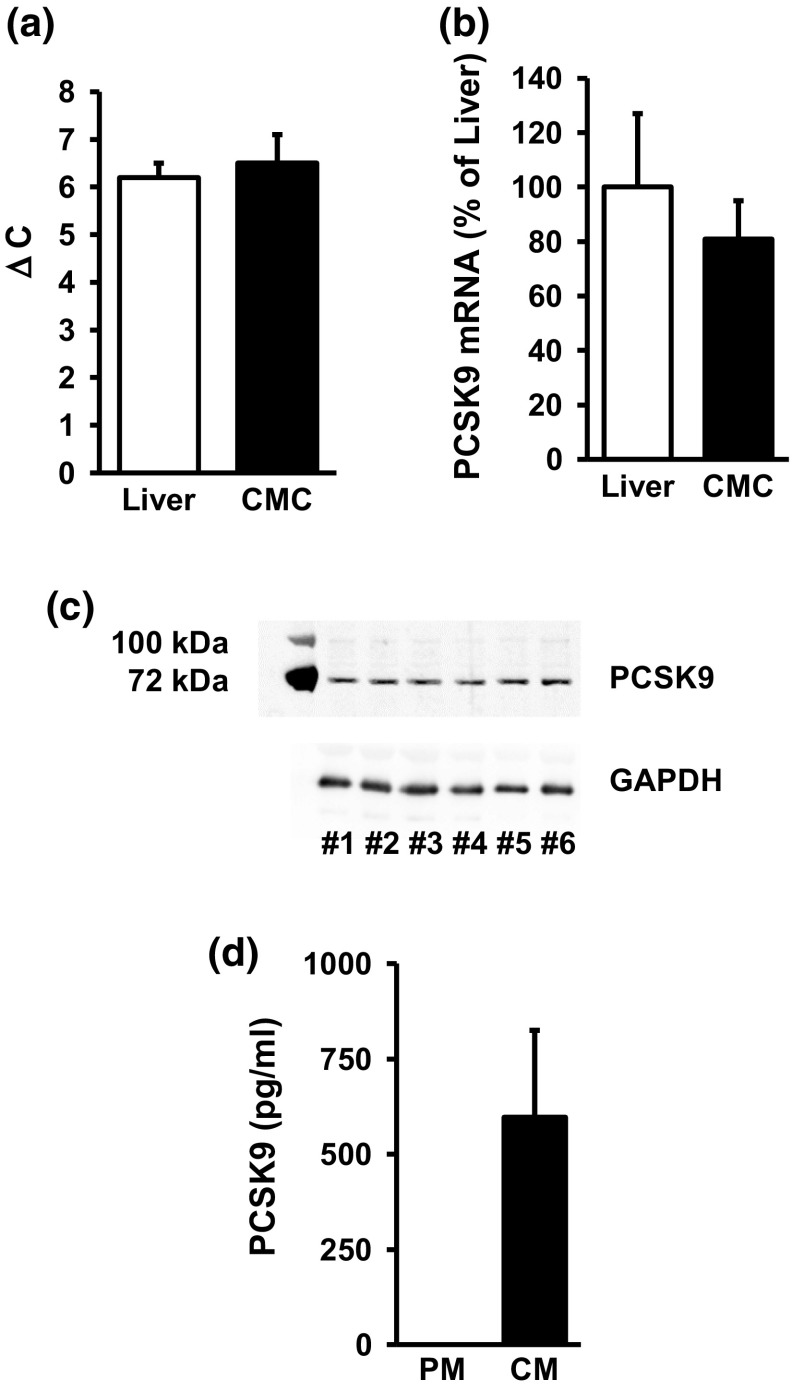
Expression of PCSK9 in liver and cardiomyocytes (CMC). **a** Cycle number for the threshold of PCSK9 amplification of the samples are given for (*n* = 10, eight independent experiments, CMC) and liver isolated from normotensive rats (*n* = 5). Data are normalized to the mean expression of the three house-keeping genes GAPDH, HPRT, and B2M. **b** Relative expression of PCSK9 in cardiomyocytes normalized to the expression in liver. All data are normalized to the three house-keeping genes. Data are mean ± SD. *p* > 0.05. **c** Representative immunoblot indicating the expression of PCSK9 (top) and GAPDH (bottom). Original blot is shown in supplementary Figure 1. **d** Release of PCSK9 into the medium (*PM* plain culture medium, *CM* conditioning medium, 24 h incubation, *n* = 6); data shown are mean ± SD from *n* independent preparations

oxLDL has been reported to increase the expression of PCSK9 in non-hepatic cells. It was not known whether terminal differentiated cardiomyocytes express PCSK9 and whether oxLDL modifies its expression. We compared the basal expression of PCSK9 in terminal differentiated cardiomyocytes with that of the liver, taken as a positive control for the expression of PCSK9. In liver samples, real-time PCR analysis depicted PCSK9 6.2 ± 0.3 cycles later than the house-keeping genes. In samples taken from isolated adult cardiomyocytes, PCSK9 appeared after 6.5 ± 0.6 cycles later (Fig. 5a). Normalized to the expression level in the liver, cardiomyocytes express PCSK9 on the mRNA level by approximately 81% compared to the liver (Fig. 5b). On the protein level, an antibody directed against PCSK9 recognized a protein of the expected molecular weight of 76 kDa in all preparations of cardiomyocytes (Fig. 5c; Supplement Fig. 4). Finally, cardiomyocytes released PCSK9 into the medium during cultivation (Fig. 5d).

**Fig. 6:**
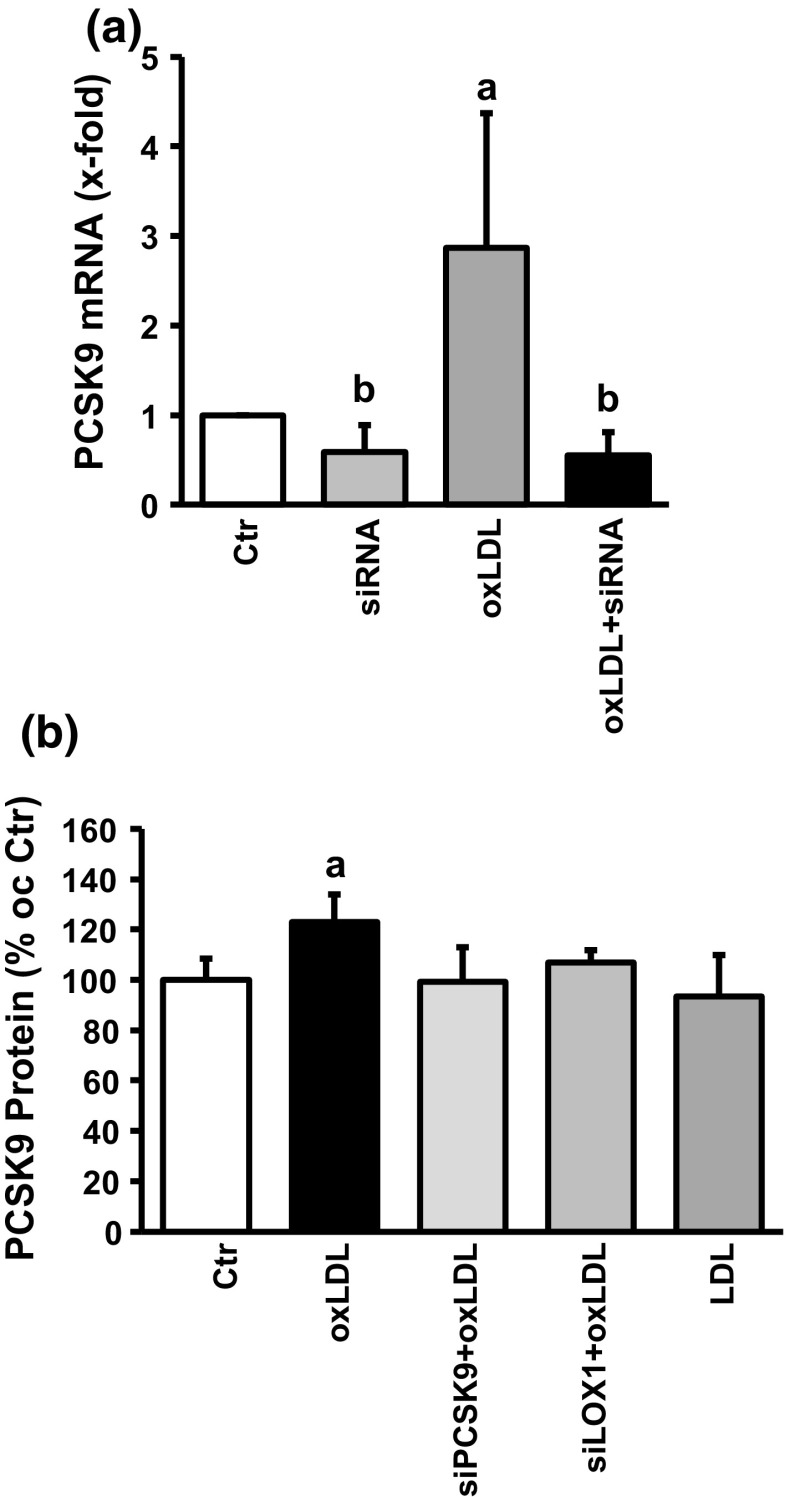
Effect of oxLDL on PCSK9 expression. **a** RT-PCR analysis for cells under basal conditions and for cells exposed to siRNA directed against PCSK9, oxLDL (20 µg/ml), or a combination of both. Data are mean ± SEM from *n* = 6–9 independent experiments; **b** *p* < 0.05 below control; a: *p* < 0.05 above control. **d** Quantification of the western blots analyzing the expression of PCSK9 in the presence of oxLDL or LDL (20 µg/ml) and siRNA directed against PCSK9 or LOX-1, or combination of them. a: *p* < 0.05 above control. Data are mean ± SD from *n* = 3–7 independent experiments. Representative blots are shown in supplementary Fig. 2

Once we identified PCSK9 as a constitutively expressed gene in adult terminal differentiated cardiomyocytes, we next investigated whether oxLDL increases the expression of PCSK9. oxLDL increased PCSK9 mRNA expression by 1.80 ± 0.17-fold (Fig. 6a) and PCSK9 protein expression by 23 ± 3% (Fig. 6b; representative immunoblot shown in supplementary Fig. 5). These data suggest that oxLDL increases the expression of PCSK9. Therefore, siRNA directed against PCSK9 was added to the culture medium 6 h prior to oxLDL treatment. Collectively, siRNA directed against PCSK9 reduced PCSK9 mRNA expression in the absence and presence of oxLDL (Fig. 6b) and attenuated the effect on protein induction (Fig. 6b). Furthermore, induction of PCSK9 protein expression could also be attenuated by siRNA directed against LOX-1, whereas LDL could not replace oxLDL in inducing the protein expression of PCSK9 (Fig. 6b).

**Fig. 7:**
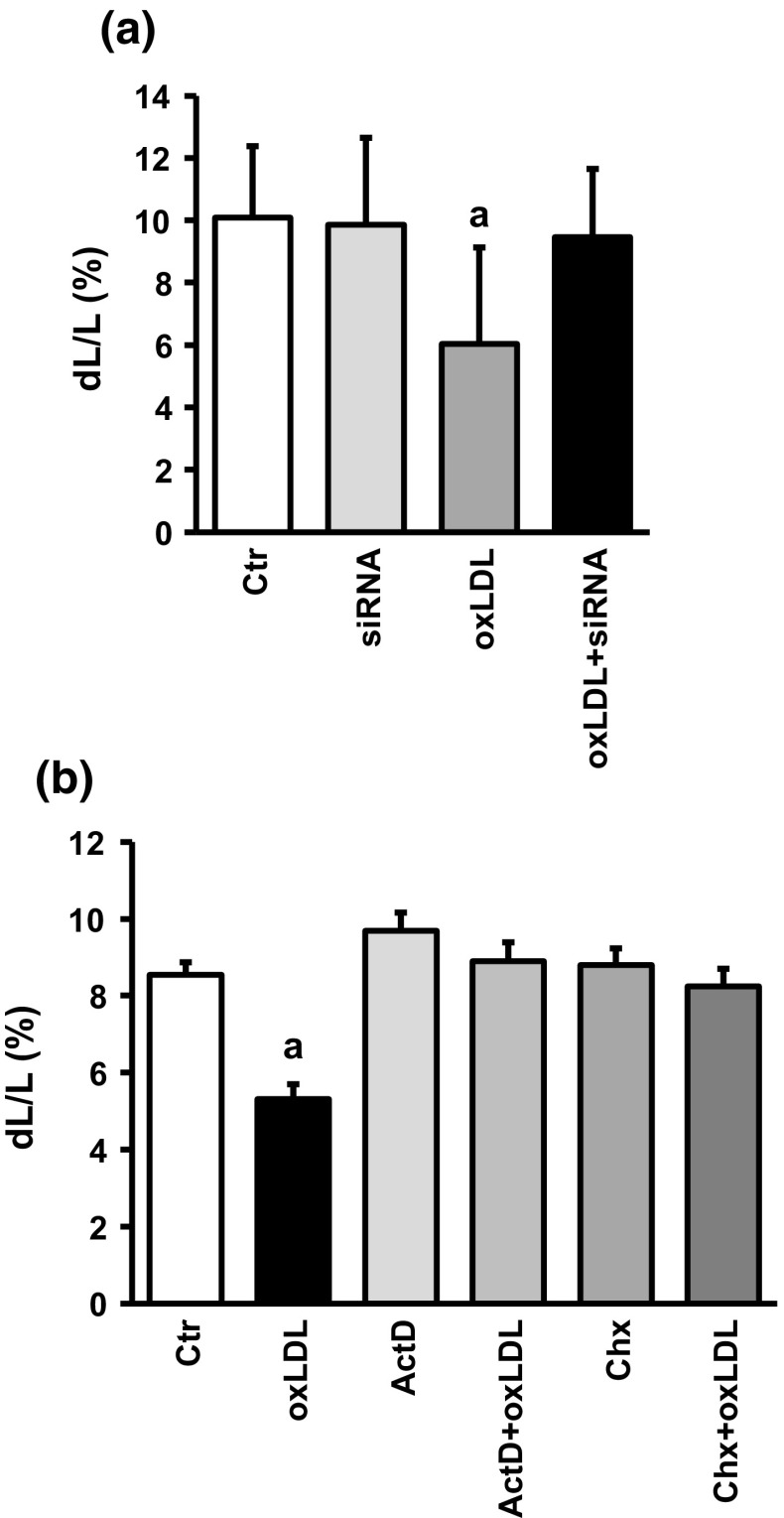
Effect of silencing oxLDL-dependent induction of PCSK9 expression on cell shortening. **a** Cells were exposed to oxLDL (20 µg/ml) or siRNA directed against PCSK9. Data are mean ± SD from 90 cells each from ten independent experiments. a: *p* < 0.05 vs. control, siRNA, and oxLDL plus siRNA. **b** Cell shortening of cells exposed to actinomycin D or cycloheximide with and without oxLDL; a: *p* < 0.05 vs. all other groups

These data suggest that induction of PCSK9 protein expression is involved in oxLDL-dependent effects on cell shortening. We finally proved this assumption by administration of siRNA directed against PCSK9 to oxLDL-treated cardiomyocytes and found that under these conditions oxLDL did no longer impair function (Fig. 7a). The hypothesis that induction of PCSK9 is required for oxLDL-dependent effects on cell shortening is further proven by addition of actinomycin D (transcriptional inhibitor) and cycloheximide (translational inhibitor) to cell treated with oxLDL. In both cases, oxLDL alone did not modify cell shortening (Fig. 7b).

## Discussion

There are three main new findings in this study: First, we show that oxLDL directly affects cardiac function. Second, we show that adult terminally differentiated cardiomyocytes constitutively express PCSK9. Third, we show that oxLDL-dependent effects on cell shortening depend on induction of PCSK9 expression. Therefore, oxLDL is not only a biomarker associated with oxidative stress, cardiac dysfunction and heart failure [21, 31] but it plays an active role in this process and PCSK9 exerts important direct cardiac effects.

Heart failure, the inability of the heart to support the body with sufficient blood circulation, has several features. On the level of cardiomyocytes, oxLDL had been associated with induction of apoptosis, alterations in generation of action potential, and induction of gene transcription that is normally linked to cardiac remodeling (BNP, MCP-1). Unfortunately, such experiments were always performed with cells that cannot be directly compared to terminally differentiated ventricular cardiomyocytes [2, 8]. Experiments that were performed on rabbit cardiomyocytes are an exception [36]. In these studies, oxLDL caused significant cell damage and membrane depolarization but authors incubated cells with concentrations of chemically modified LDL 25-fold stronger than the concentrations used in this study. In similar experiments performed on rabbit cardiomyocytes, a threshold concentration of 100 µg/ml was determined for acute effects on calcium transients. This concentration is still fivefold higher than that used in this study [15]. Similar high concentrations of oxLDL were also linked to cell damage in endothelial progenitor cells, whereas lower concentrations could be tolerated or even improve biological activity [13]. In our study, oxLDL significantly impaired cell shortening without producing cell damage as indicated by unaltered expression of genes involved in the regulation of cell apoptosis, such as bcl-2 and bax, as wells as unaltered gross morphology and the absence of apoptosis or necrosis. In contrast, exposure of neonatal cardiomyocytes to oxLDL (50 µg/ml, 24 h) caused cell death by apoptosis. In conclusion, we describe specific effects of oxLDL on cell functions that are independent of general cell damage. Of note, we had a certain level of cell necrosis and apoptosis after 24 h cultivation under serum-free conditions. This was, however, independent from oxLDL, and furthermore, in the well known range of serum-free cultured adult ventricular cardiomyocytes. Thus, there is no additive effect of oxLDL on cell damage. It is possible to reduce the level of cell damage significantly by addition of serum to the cultures but this is not necessarily by free of LDL and oxidized LDL and therefore not the first choice in experiments performed with oxLDL.

Cardiomyocytes respond to LDL particles dependent on the concentration and modification of these particles. In principle, this can be linked either to the cellular uptake of such particles or by activation of specific receptors. However, cholesterol uptake is low in cardiomyocytes and classical LDL transporters are not required to maintain a sufficient cholesterol concentration in the cells [20]. Therefore, the most likely mechanism by which LDL particles may affect cardiac function is by binding to and activation of LDL receptors. Three different receptors have to be taken into account: LDL receptors, known to act as LDL transport molecules in the liver [25], LDL receptor-related protein-1 (LRP-1), known to improve load-free cell shortening in these cells [18], and LOX-1 that has been associated with heart failure [29]. Among these receptors, LOX-1 was by far the strongest expressed receptor in cardiomyocytes. Despite oxLDL, advanced glycosylated end products (AGE), aged red blood cells, leucocytes, platelets, and apoptotic cells can activate this receptor. AGE and apoptosis are common findings associated with oxidative stress and subsequently cardiovascular disease [4]. Furthermore, LOX-1 is important for the modulation of angiotensin II-dependent hypertrophy. Angiotensin II-dependent hypertrophy leads to reduced cell function and can directly impair load-free cell shortening similar than oxLDL [17]. However, all these findings are associations but not causal links between oxLDL, LOX-1 activation, and cardiac dysfunction. Such a causal relationship was given here because silencing of LOX-1 attenuated the effect of oxLDL. Moreover, stimulation of LOX-1 is known to be associated with p38 MAPK activation in all cell types, whereas the co-activation of other MAPK pathways by oxLDL remains cell specific. In terminally differentiated cardiomyocytes used in this study, inhibition of p38 MAPK pathways but not of p42/p44 MAPK pathways or c-jun kinase pathways attenuated the response to oxLDL. On top of this we show that oxLDL directly increases the phosphorylation of p38 MAPK. Of note, activation of the stress-dependent p38 MAPK occurs by oxidative stress [34]. Oxidative stress in cardiomyocytes caused by oxLDL was also confirmed by direct oxidative modification of tropomyosin. All together, the level of expression, the attenuation of oxLDL effects by silencing the receptor, and activation of known LOX-1-dependent signaling pathways strongly support the idea that LOX-1 activation triggers the response of oxLDL in cardiomyocytes.

**Fig. 8:**
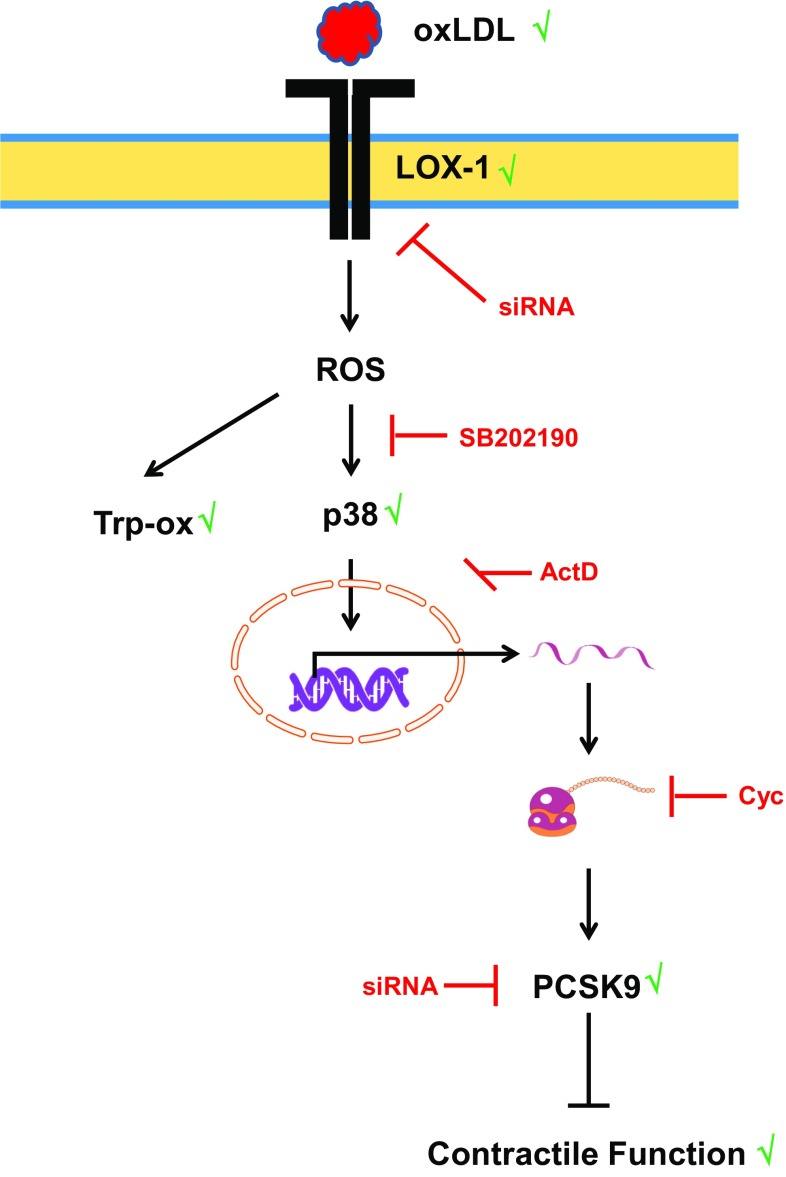
Summary figure of our findings: oxidized LDL (oxLDL) activates the oxLDL receptor. Expression of the receptor by cardiomyocytes was shown in this study and silencing of the receptor by siRNA attenuated all subsequent steps. oxLDL induces oxidative stress as indicated by oxidative modifications of tropomyosin (Trp-ox). Oxidative stress activates p38 MAP kinase as indicated by western blot. Inhibition of p38 MAP kinase activation by SB20190 attenuates this effect. Subsequently, oxLDL causes increased expression of PCSK9 as indicated by western blots and inhibition of transcription by actinomycin D (ActD) or translation by cycloheximide (chx) block all subsequent steps. Silencing of PCSK9 upregulation attenuates future steps

oxLDL increases the expression of PCSK9 in non-hepatic cells [27]. The proinflammatory response of macrophages to oxLDL has been linked to the induction of PCSK9 [27]. Furthermore, PCSK9 regulates the degradation of epithelial sodium channels (ENaC), suggesting multiple cellular targets of PCSK9 in non-hepatic cells [26]. Therefore, we addressed the question whether PCSK9 might be involved the oxLDL-dependent effects described here. Whether cardiomyocytes express PCSK9 or not has not been addressed before. We found that cardiomyocytes constitutively express PCSK9 on the protein and mRNA level. Furthermore, cultured cardiomyocytes released PCSK9 into the culture medium. Based on its mRNA expression the level of expression in cardiomyocytes was approximately 81% of that found in the liver, a classical PCSK9 expressing tissue. We could not downregulate the basal expression of PCSK9 in cardiomyocytes by supplying siRNA directed against PCSK9 within 24 h. However, even in other cells PCSK9 downregulation requires more than 24 h [3, 19]. This suggested that PCSK9 has a rather long half-life in cardiomyocytes. The antibody used here identified a rather specific protein band of the expected molecular weight (approximately 74 kDa). PCSK9 at this molecular weight is released by hepatic cells and we found also PCSK9 secretion. Furthermore, we could attenuate oxLDL-dependent increases in the expression of PCSK9 by siRNA directed against PCSK9 (protein and mRNA level). Therefore, the antibody seems to be rather specific. Furthermore, incubating the cells with cycloheximide and actinomycin D did not affect basal PCSK9 expression (data not shown). Collectively the data suggest a rather stable expression of PCSK9 in cardiomyocytes. However, in the presence of oxLDL expression of PCSK9 is induced. This effect of oxLDL was not mimicked by LDL and attenuated by siRNA directed against the LOX-1. Although the absolute increase was not very strong (approximately 25–30%), the effect on load-free cell shortening was rather strong. Whether the different assays used in this study (PCR, western blot, and cell shortening) have a different level of sensitivity or whether small increases in PCSK9 expression has a specific effect by direct interfering with up to now not identified target proteins in the cells remains to be clarified. However, future studies are required to establish downstream targets of PCSK9 in cardiomyocytes. Figure 8 summarizes our findings and experiments shown in this study.

In summary, this is the first report about long-term effects (24 h) of oxLDL on cardiomyocytes at non-toxic concentrations of oxLDL. Furthermore, this is the first report that shows stable expression of PCSK9 in terminal differentiated cardiomyocytes and describes a link between the expression level of PCSK9 and cell function. Current therapeutics targeting the PCSK9 are based on neutralizing antibodies directed against PCSK9 [25]. Therefore, current targets affect extracellular levels of PCSK9. However, with the development of antisense strategies directed against PCSK9, intracellular PCSK9 will also become a reasonable target and in this case treatment will also affect intracellular effects of PCSK9 overexpression [6]. Non-hepatic cells support only minor proportions of plasma PCSK9 but intracellular PCSK9 affects cellular function in these cells. However, intracellular targets of PCSK9 in cardiomyocytes have still to be defined and our data do not exclude a possibility that cardiomyocytes secrete PCSK9 that then acts in an autocrine way on cardiomyocytes. This requires future studies that were beyond the scope of this study.

## Electronic supplementary material

Below is the link to the electronic supplementary material.

Supplementary material 1 (DOCX 1487 kb)
