# Supplementary material for: Oxidized low-density lipoprotein (oxLDL) affects load-free cell shortening of cardiomyocytes in a proprotein convertase subtilisin/kexin 9 (PCSK9)-dependent way
Source: Basic Res Cardiol. 2017 Sep 14;112(6):63. doi: 10.1007/s00395-017-0650-1 (PMC5599470; doi:10.1007/s00395-017-0650-1)
Supplement: Supplementary file 1 — Supplementary material 1 (DOCX 1487 kb) [file 395_2017_650_MOESM1_ESM.docx]

Legend to the Figure: Cells were cultured for 24 h in the presence of scrambleRNA (scRNA; white bars) or siRNA directed against LOX-1 (black bars). Subsequently, cells were harvested as described in Material and Methods and protein samples were blotted. Blots are incubated with an antibody against LOX-1 (GeneTex, GTX59636). The antibody recognized two bands: The approximately pro-form of the receptor (52 kDa) and a smaller unspecific band (45 kDa). The pro-form was reduced in both preparations. The mature form (35 kDa) was found in liver preparations but the expression in cardiomyocytes was below the detection level.


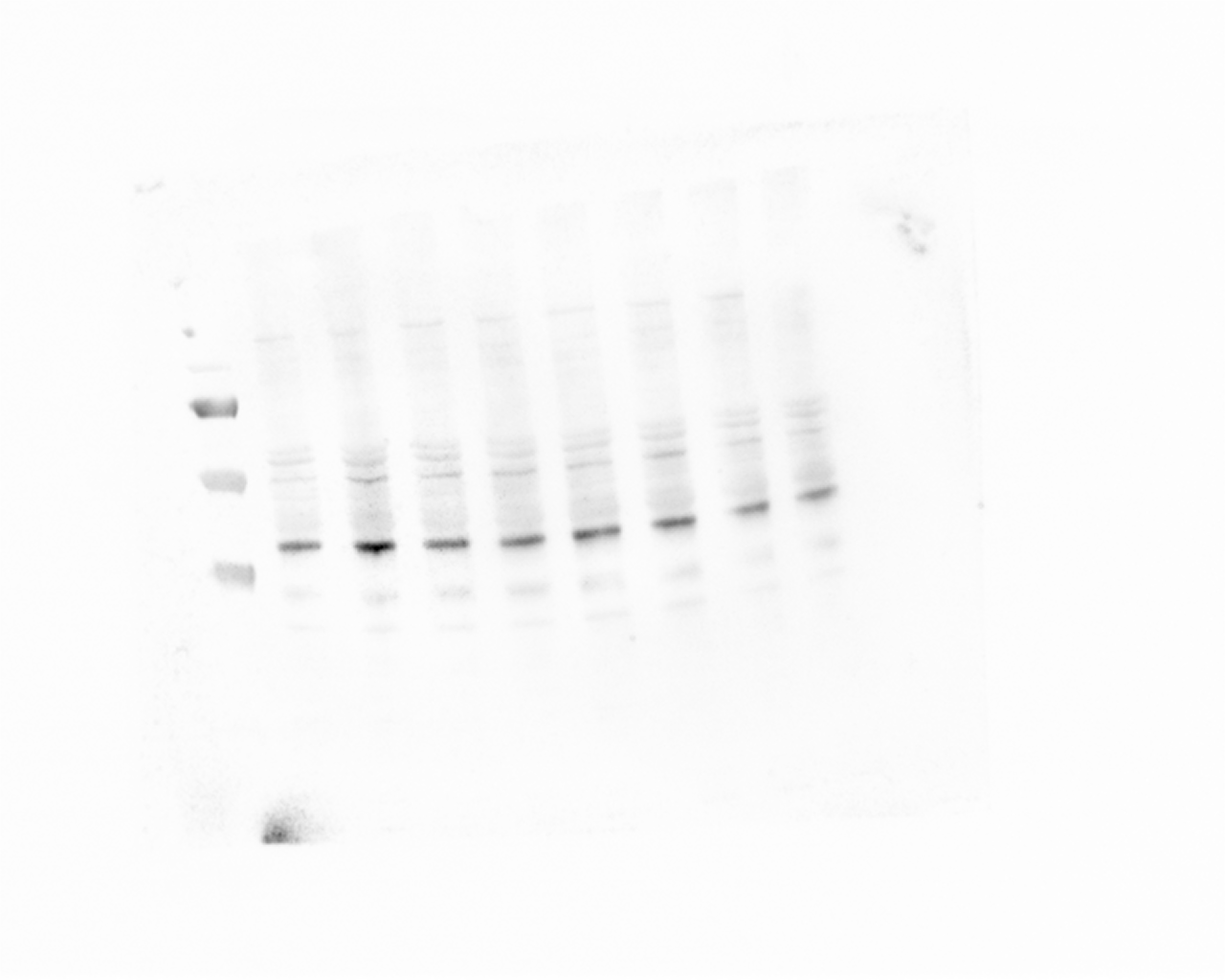


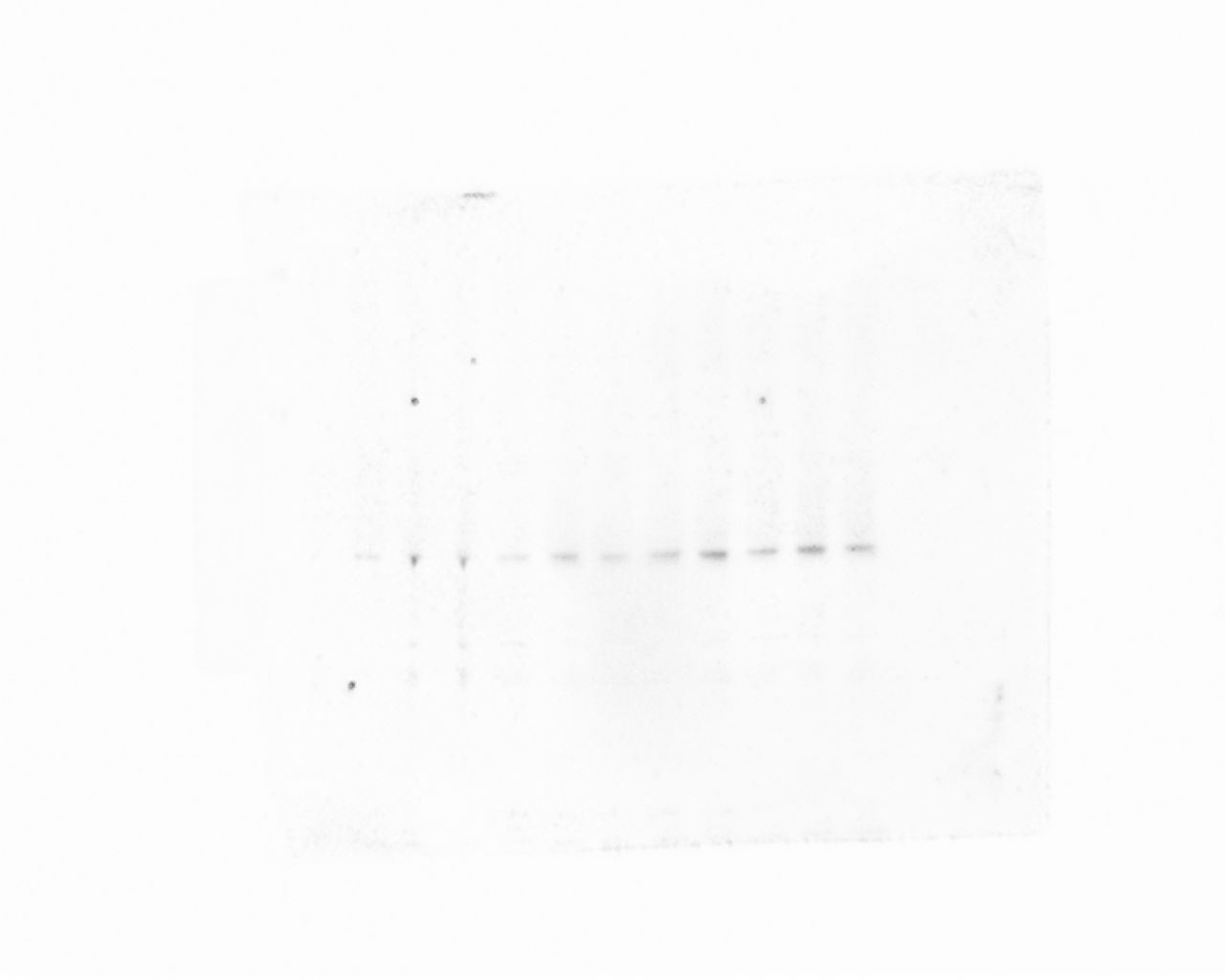


Supplement Fig. 2: Original blots for p38 MAP kinase activation (see Fig. 4). Top: Non-Phosphorylated; Bottom: Phosphorylated.
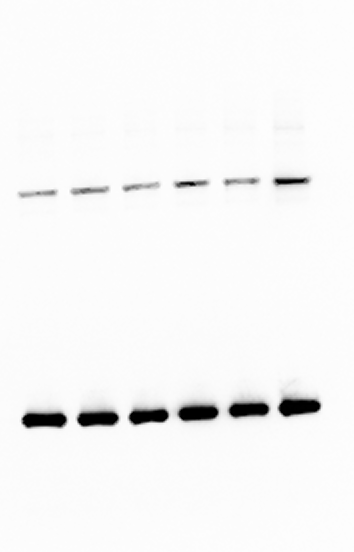





Supplement Fig. 3: Original blots for Tropomyosin and its oxidative modification (see Fig. 4).


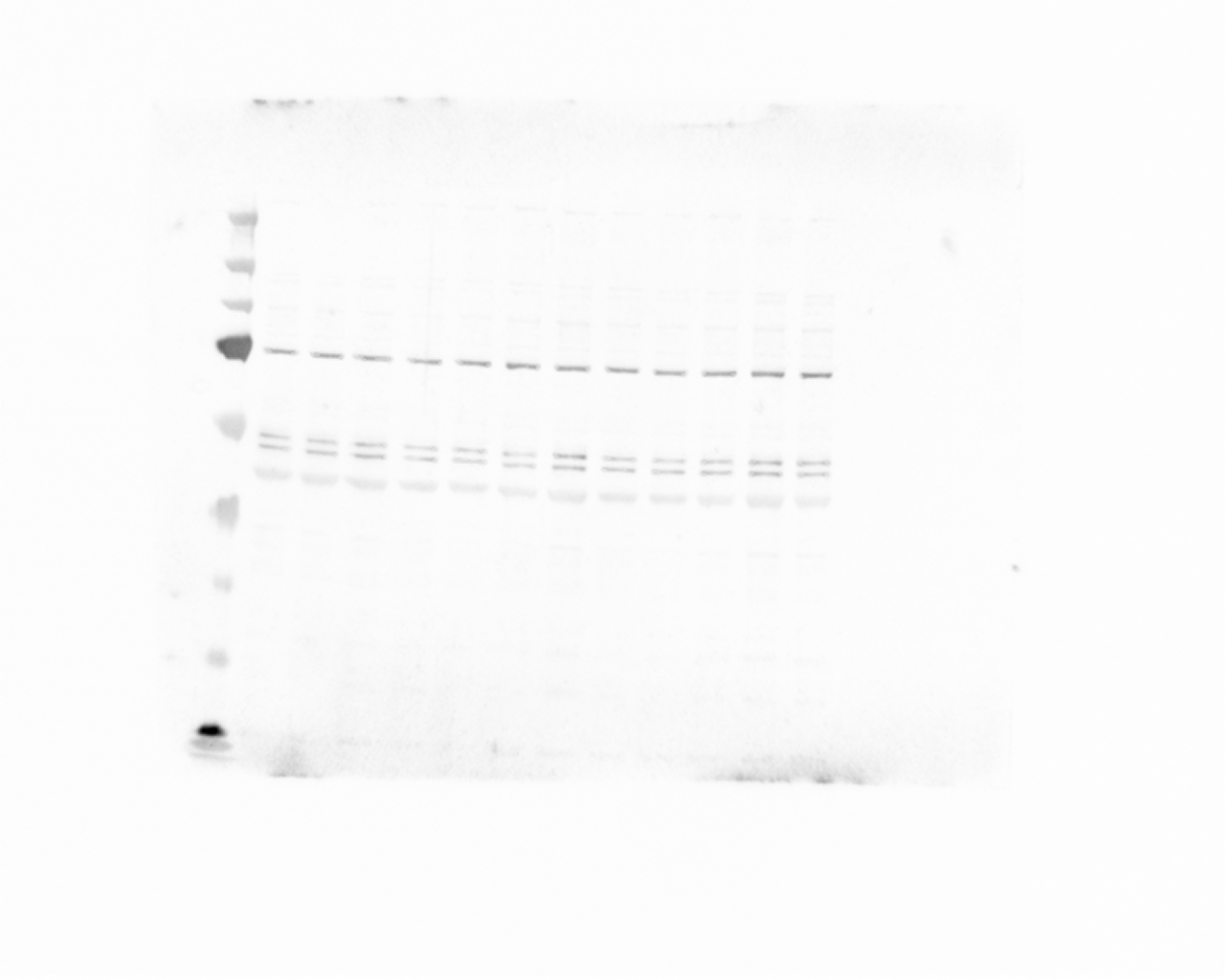
**Suppl. Fig. 4**

**Oringal blot of PCSK9 expression in cardiomyocytes. See figure 5.**

**Suppl. Fig. 5**

Representative blots showing changes in PCSK9 expression in individual experiments.
